# New York Odontological Society

**Published:** 1895-02

**Authors:** 

**Affiliations:** New York Odontological Society


### Reports of Society Meetings.

### NEW YORK ODONTOLOGICAL SOCIETY.

A REGULAR meeting of the New York Odontological Society was held on Tuesday evening, October 16, 1894, at the New York Academy of Medicine, No. 17 West Forty-third Street, New York City.

The meeting was called to order by the President, Dr. Brockway, who welcomed the members after the summer vacation.

The Secretary read the minutes of the previous meeting, which were approved.

### INCIDENTS OF OFFICE PRACTICE.

*Dr. Jarvie.*—About two years ago a lady called on me, complaining of severe pain in the right superior lateral. The tooth had never been filled, and was then free from decay, yet pain in it was frequent, and it was particularly sensitive to thermal changes. After treatment for some days and the pain not subsiding, I drilled into the palatine surface and applied creosote and arsenic to kill the pulp. After the removal of the pulp, and upon pumping carbolic acid into the canal, I noticed carbolic acid oozing out between the gum and the root upon the palatine surface. Close examination revealed absorption upon that side of the root reaching inward to the pulp. Further examination revealed a corresponding cavity of absorption upon the labial side of the root, reaching into the pulp-canal. The points of absorption were about one-quarter the length of the root beyond the gum margin. The tooth was treated and filled.

During the past summer the tooth was broken off, the fracture occurring at the point where absorption had taken place and where the strength of the tooth had necessarily become much impaired. I was away at the time, and as the broken part was still attached slightly to the gum, Dr. Turner, my associate, tied it in place to the adjoining teeth with waxed silk until my return.

This seemed a case for transplantation, as for many reasons it was undesirable for the lady to wear a plate. After failing to secure a suitable natural tooth, I got a lateral with badly-decayed crown, but with a good and suitably-shaped root. To this root I attached a porcelain crown that matched the natural one perfectly, but I

was perplexed for a time to know how to remove the portion of the root remaining in the jaw, which was firmly held in the socket. Any ordinary means would bruise the gum and possibly fracture the socket, and induce a condition of affairs fatal to a successful transplantation. I finally drilled through the centre of the root, almost to the end, following the canal, with a Gates-Glidden drill; cut a thread in this, and then inserted a screw firmly, pulling out the root by this means without injury to gum or process. I then inserted into the socket the root with artificial crown attached. This operation was performed ten days ago, with what ultimate success time alone can tell.

*Dr. Lord.*—I may say that in my experience in using gold and tin together I get better results from having the gold very largely in excess, and folding the two foils together; then, if I want at any time to make a filling so as to neutralize the color of the gold, I put about one-twentieth of tin with the gold, and it makes a very pretty color, and one that will not tarnish. It is very nice for the labial surface.

I may also say that Mr. Williams has recently brought out some tin-foil that is very much superior to any that I have ever used. I have said at our meetings that the tin that I procured from him some twenty-five years ago I thought was better than any that he had made since, or any that I had ever seen. He has overdone it this summer, however, and his tin is superior to that which I thought was as good as it could be. It works waxy, I might say; we can do anything with it, with suitable instruments. A very little of it put with soft gold makes the gold tougher. It requires much less anchorage, and you can build out or contour with it as much as you desire, if the cavity has three walls remaining.

*Dr. Watkins.*—Would you use very thin foil?

*Dr. Lord.*—The foil is thinner than that which I have used for many years, although it is not what we would call thin. That which I used previously was marked Number 4, but I think it was a heavy Number 4. This is Number 3. I said to Mr. Williams some time ago that I wished he would get out a thinner foil, and he did so; but it did not compare with the foil which he has now produced.

*Dr. Perry.*—Many years ago, when there was a craze in reference to the use of tin-foil at the cervical margin with gold covering it, I indulged in the practice for a few years, but I have not done so for a long time, for I found that the tin-foil in time dissolved. I also used the gold- and tin foil rolled together in different proportions, but I do not do so now. With a good quality of soft gold, I fail to see the real advantage of combining tin with it.

In reference to the case which Dr. Jarvie has reported, I would like to ask him if the tooth itself could not have been placed on a suitable root, the advantage being that if it were a reasonably sound tooth a more natural appearance would be secured than by an artificial tooth. I have put artificial crowns on natural roots a great number of times, and with great satisfaction, because it is easy to secure the proper size and length of crown; but I always think an artificial tooth is not like a natural one in different lights,—in the evening, for instance.

In reference to getting rid of the root, Dr. Jarvie's method is a very ingenious one. Once when I had a root which I could not get out without fear of injury to the gum, I patiently and deliberately drilled the root all away. It was a very tiresome operation, and I should not like to do it again. It was done without pain to the patient.

*The President.*—Could you not have removed it with a screw?

*Dr. Perry.*—It was so far gone, and the opening through the root was in such a condition, that it seemed to me I could not get a secure enough foundation for the thread of the screw to warrant my doing so. This question, of course, brings up the subject of implantation. I recently had a young lady patient with a superior left second molar from which great trouble had arisen many times during the last two or three years, to the point of wearing out her patience and exhausting my own in the way of trying to keep the tooth filled. I finally said to her that I thought she had better have the tooth out. She went to Dr. Hasbrouck and had it extracted, and when she brought it to me, I found a large amount of abnormal tissue between the three roots, a condition which clearly explained the fact that no filling could be kept in the tooth for any length of time. I cleaned that abnormal thickening of the periosteum all off very nicely, filled the tooth to the extremities of the root, put it back for her, and secured it in place by drilling a little hole through the edge of a filling on the wisdom-tooth adjoining, and passing a thread through that, and carrying it across the end of the molar, and then tying it around the first molar to keep the tooth from slipping out at night. Yesterday I took off that thread, and now the tooth is seemingly as nice and comfortable as any other tooth in her mouth. The operation was performed a week ago last Thursday. She asked me what I could promise in reference to it. I said, "I will make no promises. It is worth the trial, because the trial is made easily. It may become ankylosed. I have never implanted a tooth in that condition before, so I cannot say." It looks very favorable now, and is thoroughly comfortable. It was a little painful the first night, but nothing to compare with the pain before the tooth was extracted.

*Dr. Watkins.*—A few years ago there was considerable discussion on implantation, and there was a difference of opinion as to whether the periosteum should be removed or left when the tooth was implanted. Some removed it, others did not. Some went so far as to claim that the percentage of failures depended on whether the periosteum were perfect or imperfect. I would like to ask Dr. Jarvie and Dr. Perry what their experience has been in regard to that.

*Dr. Jarvie.*—I have not had so many cases of implantation as some others have had. I prefer always to use a root with the periosteum on it, and I take care not to disturb it if possible. I do not say it is necessary to have the periosteum intact, but I prefer to have it. In regard to the case which I related, I did think of using the natural crown of the tooth, but I was able to get a porcelain one that so closely resembled the natural one that I think it would be difficult for any one in the room to say which was the porcelain crown without the aid of a mouth-glass. As the porcelain would be stronger and sure to be free from decay in the future, I thought it wiser to use it. I had at first intended to take out the root in the way Dr. Perry mentioned, but the thought of the screw afterwards came to me, and it left a very clean, unbruised socket, which I considered very desirable.

*Dr. Perry.*—In reference to Dr. Watkins's question, I should be willing to venture this opinion: that it does not make much difference as to the presence of the periosteum. I thought in the early days that it was necessary to have it, but it is hard to find a tooth with the whole periosteum in good condition. I do not care so much whether it is on the tooth or not. After having made many trials, I have come to be more careful in reference to the age of the tooth implanted than the condition. I consider that young teeth are not so favorable for implantation as older ones. I think they become absorbed more readily. My view is that an old, hard tooth is more favorable for implantation.

*Dr. Jarvie.*—How about the fit in the socket?

*Dr. Perry.*—A good fit in the socket is valuable for the beginning, but I do not think it essential.

*Dr. Jarvie.*—I prefer not to have the tooth fit very tight into the newly-made socket. I think the best success I have had is with a tooth I inserted about seven or eight years ago. A gentleman had a second superior bicuspid that was very loose from pyorrhœa. The attachment was almost entirely gone. I removed the tooth, and with a large bur cut away all affected bone and considerable of the soft parts, making a socket that was as large again as the root of the tooth that I put in. I placed the tooth in position and tied it with silk ligatures, which is the method I always employ, and I think that tooth is the firmest one in the gentleman's head to-day. That may not be saying much, as they are all rather shaky.

*Dr. Watkins.*—Are you particular to use a tooth which has been preserved in an antiseptic from the time it was extracted, or do you make use of any tooth which you find?

*Dr. Perry.*—I let them dry up, though I do not like to have them crack. I used to keep them in a bottle with a small, moistened sponge. Now I simply soak them in bichloride for a short while before I use them. I have had over a hundred cases of implantation; I do not think more than one-half of those cases are in to-day. But I think more than one-quarter of them are very handsome successes. How long they will be I do not know. Often when a tooth comes out I slip another one in. I have done this as many as four times in the same socket, ranging over a period of six or seven years.

*Dr. Hodson.*—I would like to ask Dr. Perry if he is not particular about there being any "bark" on the root,—what about the porcelain ones that we heard about a year or so ago?

*Dr. Perry.*—That has been tried and given up.

*The President.*—I see that Dr. Sailer has brought something for us to-night. I hope he will explain it to us.

*Dr. Sailer.*—It is the custom of the profession to-day, rather contrary to what was done some time ago, as I understand from the older members of the profession, if they think they have anything new, to present it for the benefit of their brethren. I have been using this instrument in a more or less perfect state for two years, and several of the gentlemen have seen it and desire me to present it to the Odontological Society. The first consideration for a dentist to adopt anything in his practice is, whether it adds to the perfection of the operation, the ease and comfort of the patient, and comfort and ease of the operator. I think this little thing, which I call a "combination box," because it contains seven different things, adds to all three of these elements. This box is divided into two compartments on this side to hold water. There is a sterilizer, in which the water can be kept at a temperature of from 180° to 212°. There is a compartment to be used in case you want an impression with modelling compound. You have it right at hand, and there is no delay. There is a little wire frame for holding the mirrors. It keeps them at a temperature which prevents the breath from condensing on them. There are two little compartments for gutta-percha. There is another compartment for gutta-percha which does not require such a high degree of heat. The gutta-percha can be kept there for days at a time without losing any of its qualities. There is a glass of water, which is held in place by springs at the side. It permits of the circulation of air around the glass. You can have the water in the box at 190° or 200°, while the water in the glass is at a much lower temperature. There is a depression here to hold the instruments for using gutta-percha. If you wish to introduce gutta-percha into a tooth to destroy a pulp, or where the pulp is nearly exposed, and you do not feel justified in covering that tooth with the oxide and phosphate, as we sometimes do, but want to wait for results, you can introduce gutta-percha into cajuput, and use the two materials together. You can introduce it at a warm temperature and it will be more effective. There is a little slab here to hold medicines for a sensitive tooth. I introduce them warm, and I get as a first result the soothing and warm effect, rather than the pain which comes from the use of a cold medicine. This box is placed beside my cabinet. In my office it is on a bracket, about six or eight inches long. It is always at my right hand, and always ready for use. I have found it a great convenience for the last two years.

*Dr. D. W. Barker.*—Your President has requested me to show you an improved method of disinfection of putrescent pulp-canals. The materials which have been introduced for that purpose within the last few years contain a surplus of oxygen, which, when brought in contact with any material which has a strong affinity for oxygen, as pus, for instance, causes the pus products immediately to change their nature. These materials, however, have some disadvantages. They are almost all extremely caustic. That objection applies to the strong sulphuric acid which is now much used. The materials which I prefer for that purpose are two very simple ones, and by their union in the pulp-canal they set free the nascent oxygen. They are permanganate of potash and peroxide of hydrogen. When brought in contact in the canal the effervescence is very violent. I will illustrate it in your presence. The best way to do it is to take a Donaldson bristle and dip it in the powdered permanganate of potash, and then with a little syringe drop a little peroxide of hydrogen on it. [Dr. Barker illustrated the effect of the two materials on his hand.] It produces on the hand only a slight warmth. If the two are made in a solution the union is much more violent, amounting almost to an explosion, and the effect is much more noticeable. It does not stain the teeth. I have stained my hand because I did not use a sufficient quantity of the hydrogen to neutralize the potash. I have been using it for some little time. The teeth so treated show a remarkably quick cure. I have used this method, and after two or three applications the tooth fails to show the characteristic foaming when peroxide alone is introduced. I have attempted to close up and fill them in a week or ten days, just to see how quickly it could be done, and with very good success.

*Dr. Hodson.*—Does the formation of gas produce any irritation to the periosteum?

*Dr. Barker.*—In my experience, I have not met any such cases.

*Dr. Hodson.*—Are you particular to have it go to the end of the root?

*Dr. Barker.*—I have not exercised any precautions to prevent that. As soon as I have the peroxide on the powdered potash, I introduce a Donaldson bristle, and pump it up and out quickly.

Dr. Davenport then read a paper entitled "The Correction of Injury resulting from Extraction," after which the President called upon Dr. Bonwill, of Philadelphia.

(For Dr. Davenport's paper, see page 81.)

*Dr. Bonwill.*—I do not know how many may have read my article after it was published. I know of several gentlemen who did not read it; they were honest enough to confess that to me. I thought I was condensing the whole thing as much as possible, but I will recall the principal points, since so few have perused the essay as published.

[Dr. Bonwill then read a *résumé* of his paper.]

1. My "New Era" is not the "New Departure" in any way. It is adaptability, not compatibility.

2. The title is an assumption based on a practice from varied experience, precedents, and results.

3. I assume that the hour has arrived when we must adopt some practice that will more generally attain the ends for which every dentist in the land cries out. That there is a void in everything done by us that no system, now extant, can fill.

Our failures.—1. The majority of men who enter our ranks.

2. Even with good men failure is with us on every side. The journals and all essays and discussions attest it.

3. The materials.—When and where to be used, and how to manipulate gold, amalgam, tin, oxyphosphate, gutta-percha.

4. The instruments for placing that material into cavities formed by art.

5. The poorer class of teeth upon which we operate every year growing worse.

6. Can human teeth be conserved better if the subject be taken at the third or fourth year, and have a larger number of teeth saved by other materials than the use of gold and the metals?

7. Are we not doing irreparable mischief to our patrons by the use of so much gold, in the early years, up to the twentieth?

8. How far can we go in anticipating caries and have the average dentist do it?

9. How far after superficial decay has started are we justified in attempting its arrest without filling with anything?

10. How far can we ignore self in order to reach our highest aims as a profession to which we have pledged our lives, talents, and sacred honor? To do to the public as we would do to our own families?

11. Have we not in attempting to reach the most perfect results used too many kinds of gold, amalgam, oxyphosphate, and gutta-percha in the same mouth.

12. Failing in all, have we not resorted to instruments that will, like the boomerang, come back to us?

13. Are we doing the proper thing in abandoning filling for a substitute of crowns and bridges, making of filling and saving human teeth a "lost art"?

14. With every appliance, instrument, dam, filling-material, and brains, yet the cry is failure on every hand; you see it in every journal; it is a principal topic of the hour!

15. No better indication of failure than the craze for copper amalgam, mixed fillings, gold and tin, gold and amalgam, amalgam and oxyphosphate, and even gutta-percha, to prevent recurrence of caries at the cervix!

16. I told you of the ignorance extant of not knowing the laws of articulation and the true mechanism and function of the human teeth.

17. I told you of the utter failure to keep the first permanent molars as far back towards the ramus that the arch may attain its normal size, symmetry, beauty, and usefulness.

18. I told you of the sin of cutting indiscriminately the approximal surfaces of the teeth, and flat fillings and fillings not enough plus contour and false articulation, the result, and how retrogressive steps in and the heredity is tainted.

19. I called your special attention to my sheet-anchor in this holy war against caries and irregularity; of the manner in which I use pink gutta-percha, not only as a permanent filling in the temporary but the permanent teeth, and how I gain the full plus separation for proper contour.

20. I told you how little occasion I have for conservative treatment of the dental pulp.

21. How pyorrhœa alveolaris never invades my domain with original patients.

22. I told you, also, how I make it a first and principal active factor in the treatment of pyorrhœa alveolaris, and by recognizing the laws of articulation.

23. I told you still further that with it all I am still not satisfied that I have done all I might, because I cannot have command of the environments and circumstances, nor can I reach but a short distance from my centre of action.

24. I told you I was swinging the pendulum as far as I could beyond where I wanted it to go; that some one would have the courage to do the same thing. It will thus come to the regular beat and more perfect time (operations) be kept.

25. I told you what I do for gold and oxyphosphate fillings to hermetically seal them and make more lasting.

26. I explained how we can in our labors make art more our study and do as nature always seeks, the nearest way to accomplish her purposes.

27. How we can make art conceal art.

28. I tried to impress you with the importance of having greater spaces for contour between the molars than the bicuspids, and less with incisors. That it is to permit the alveolar processes grow up once more to the cervix, to have the gum attach itself, and there prevent any pocket for food or secretions.

29. And yet I pray, as does the Freemason, for more light.

30. I felt I had a right to assume from forty years' varied experience, with a long line of precedents, the association with inventions that the world everywhere has adopted; and, above all, the desire to unselfishly do for not only the public, but for the benefit of dentists everywhere.

31. With all this, you cannot expect me to do any more than give you the results of my hourly and daily experience and intercourse with a larger number of practitioners in dentistry and medicine than few others in our ranks have enjoyed. It is not for you to antagonize what is given; not to claim it for yourselve that you have done the same thing. You must lose the credit if you do not make it known on the house-top when you first did it. Do not wait until another promulgates it.

32. Now I am through; go on, please, with your criticisms, but remember, that if I have failed to do the best by you, you must, while criticising, do more and better, and show your work in a clinic and not place it under a bushel.

DISCUSSION.

*Dr. Lord.*—It is exceedingly unfortunate that our good friend Dr. Bonwill is not able to hear the speakers. He has come here to listen to the discussion, and I would suggest that it be so arranged that he sit as near the speakers as possible, so he may hear as best he can. I feel quite a little embarrassed on account of the reading of what might be called a second paper. I proposed to discuss the paper that was read at a previous meeting and published, and this has gone over much more ground than we had before, and there are a great many things in what has just been read to us that we did not hear of before. It is to be hoped that the paper will be very thoroughly discussed by those present. Dr. Bonwill has very freely given his views; I hope the gentlemen will do likewise. I do not like to speak first on the subject; I would much rather that some one who is more able should do so. I have written what I have to say, and with your permission I will read it.

I confess that I am a good deal at a loss to know what to say about the paper, particularly in regard to some of the statements contained in it. It was said by Dr. Perry, when the paper was read, that there was good in it; but I think that we want more especially to speak of that which is bad or not so good, and let the good take care of itself. It is always well to commend whatever is good.

The paper shows interest in the subject of progress, and yet many of the points are not clearly or definitely stated; but as the author of it is an enthusiast, he is more or less excusable for some mistakes; indeed, we had better make mistakes now and then than to say or do nothing.

A great deal of ground was gone over, but most of it was not new either in theory or practice; so it is not easy to find where the "new era" comes in; and some of the statements, if taken literally, are contradictory. For instance, it is stated that "gold is a failure," and also that "gold, in itself, is good for preserving teeth." Now, perhaps it was meant that gold is a failure when it is not properly used, or where it ought not to be used, and also that it is a good material when used just when and where it is proper to use it. The statement that the "use of tin and gold in combination is another proof of the failure of gold when used alone" is very wide of the truth and facts of the case as understood by those who use these two metals together. There is one short sentence in the paper that struck me with great force as eminently true and valuable, and it is this: "There is no greater error made than to suppose it is necessary to have various qualities and forms of this metal." It has seemed to me to be most unreasonable that so many forms of gold should be called for and put upon the market, as it cannot but be misleading to the inexperienced, and is wholly unnecessary, in my judgment, in order to secure the best results.

That too much importance and value are given to the use of plastics in the paper, as they are thus far developed, would seem to be unquestionable, as well as inconsistent with sound reasoning and experience. The views are much in the wake of the "new departure creed," which has, in my judgment, greatly depreciated the art of dentistry.

The very high value put upon amalgam as a filling-material by the author of the paper is counter to the views of a very large majority of the profession. The question of the use of this material in the treatment of decayed teeth was discussed last May by the Illinois State Dental Society as the subject was never discussed before, and by some of the very best men we have; and the consensus of opinion and judgment was that it should only be used in extreme cases. That amalgam has its place as a filling-material is not questioned, except by perhaps one in a hundred of the dentists of all the land; but to recommend and use it as the "sheet-anchor," discarding (at least in a great degree) the use of foils, is something that the strongest language is too feeble to condemn. I need not again say much about the reason or reasons why amalgam is *not* so good a filling-material as foils of tin and gold. Of course good fillings made of amalgam will do better service than those that are bad; but the rule is that they change in some way or from some cause,—the margins become imperfect either from expansion or shrinkage, and leakage is the consequence,—so that the objection to its use is in the material itself; whereas if the fillings of gold- or tin-foil are made perfect, there will be no change in them, or no such change as occurs with amalgam.

It may be said that all dental operators cannot make perfect fillings of either of the foils. I do not understand that this is the question under discussion. But I believe that if amalgam had not been used to the extent that it has we should have many more perfect operators. And I will take the liberty of saying that my belief is that dental students should not have anything to do with using amalgam until towards the end of their last year at the schools. They will, in such a case, be much more likely to get a better knowledge of how to use or pack and condense either of the foils.

That which is said in the paper on preventive treatment I must regard as showing as great a difference of opinion among us as there is in regard to filling-materials. It is a most interesting subject, and one that has not received due attention as yet; but the world advances, and we may expect better things in the future.

There is one point in the paper that I do not altogether like to speak of; yet our good friend Dr. Bonwill has used great freedom in speaking of his brethren,—of their qualities and qualifications,—having the courage of his convictions, which is quite commendable in any one, as we must have convictions and stand by them. So I trust that he will not be offended at a little personal allusion, which is, the assumption of so much superior knowledge and skill.

*Dr. Perry.*—I must apologize in all sincerity to Dr. Bonwill for not being able to discuss his paper, although I intended to do so. I was in Europe when it was published, and when I came back I found my hands more than full, and since then I have not had a chance to read it. I tried to do so to-night before I went to dinner, but a number of suffering patients came in, and I had to attend to them. As far as discussing the *résumé* is concerned, it is hard to do that. Dr. Bonwill has over thirty-two points in it; one for each tooth; he has been forty years in practice, and I thought when he was reading it that he would give us one point for each year.

Dr. Bonwill, we are glad to see that you do not make great wide spaces as formerly. We are glad you make full contour, and that you do not anticipate decay any more. We are glad you do not use gold for children's teeth, but we are sorry you do not discriminate more and say you use some of the metals for them. We are glad you use gutta-percha, but we are sorry you use so much of it and stuff it so much between the teeth and into tooth cavities. It is easy enough to wedge teeth apart in two or three days and fill them, and have it done and off your mind. When you stuff gutta-percha in and let the patient bite on it for six minutes, the teeth get beyond control and become disarticulated. If you use it for young people, and make the distinction for young teeth, we will not complain at all. We will be proud of you for having the courage to tell of it. We are glad that you are coming around to the full contour. We are glad to see you advocating amalgam, but we are sorry to see you, of all men, using it where gold could be used.

If I remember rightly, the other evening you started off with a general proposition that gold was a failure. It is not! It is a grand success. It has made a success of our profession. It is the best material to-day for filling teeth in most cases. Instead of making failures throughout the profession, we are making great successes. We are doing better work every year, and doing better work through your efforts, and by the things that you have invented and taught us to use and to know. But we do not like to have you make such unqualified statements.

*Dr. S. B. Mills.*—I think what Dr. Perry said was absolutely true. When Dr. Bonwill said gold was a failure, I believe he was right from the stand-point that he uses it. Dr. Perry was right from his stand-point. But there is a golden medium. Dr. Flagg, who was very much misunderstood, made the statement that, in proportion as a tooth needs saving, gold is the worst material to use. The reason is this: I suppose I am safe in saying that seven-eighths of the teeth we are called upon to treat are of poor structure, and a large number of teeth are made useful in which we would not think of using gold. A large proportion of the fillings that are made to-day by dentists are in teeth of poor structure. Taking the mass of the profession, they can do a larger proportion of useful service to the public with the use of the combination materials, with the expense of less energy to the patient and operator, and far less expense in money. You all know I have been an enthusiast in the line in which I work; but, if I were to live my life over again, with the experience I have had, I would change my course of practice. I have spent the energy that my patients never should have lost, and spent my own energy also, and sometimes with not the best of success. I have been forty years in practice, and I have made observations in this matter, and I have come to the conclusion that there is a golden medium in practice. Amalgam, as it can be used in the hands of men who have the ability, and with the materials that we have now, is of great advantage to both operator and patient.

*Dr. Jarvie.*—I do not know that I have anything special to say, but there are a few things that I would like to give voice to, both from my recollection of the paper and from what has been said to-night. One of them is the necessity for more conservatism in statement and practice. We all know that Dr. Bonwill is an enthusiast. He enters into almost everything he says and does with such a degree of enthusiasm that his statements and actions are likely to be extreme, and they impressed me as being so when I listened to his paper. As I remember the paper, in the early part of it there was severe denunciation of the incompetency of dentists generally, stating that vast numbers of failures were constantly seen in the mouth. I do not think the failures we see in the mouth are always, or nearly always, to be laid to the incompetency of the dentist, or always to the material that is used in trying to save the teeth. A large number of teeth that come to us decayed commenced to decay quite early in life. Those teeth originally were perfect. The environment of those teeth was such that decay set in. Let those cavities be filled ever so perfectly, and with the material best fitted to the purpose, after they are filled they are not so perfect as the original unbroken surface of enamel. Let the same environment continue and the constitutional conditions remain unchanged, and those same teeth are almost sure to decay again near the former points of decay, and owing not to any faulty operation. It is the most natural thing in the world for this to occur, and the marvel is that we do not have decay more frequently near to where cavities have been filled. We must use good judgment in the selection of filling-materials. From my own experience, and from what I have seen of the operations of other men, I believe that in two-thirds of the cavities which we fill in permanent teeth gold is the best material to use. I believe that the tooth-substance takes as kindly to the gold, in this proportion of cases, as any other material, and the absolute failures in the operations where gold is used are due more to mal-manipulation than to the gold itself. A failure in an amalgam filling may not be due to improper manipulation, but to the material itself. It hardens by crystallization, and the consequent change of form makes the result uncertain, even with the most careful manipulation. With gold, it is not the material that is faulty, but it is the manipulation of it. This was a very pertinent distinction made by Dr. Lord in his paper in regard to the filling with gutta-percha between frail teeth. The gutta-percha serves two purposes,—allowing the surface of the dentine to harden under it, and the attrition upon the gutta-percha causing an enlargement of the space between the teeth so that a permanent metallic contoured filling may be put in. I believe this is a very good practice in many cases, particularly with the teeth of young people up to eighteen years of age. I have done it for years, and I continue to do it. In case of approximal decay in frail teeth, I fill cavities and space solid with gutta-percha and allow it to remain for one, two, three, or four years, according to circumstances; the circumstances being the health and strength of the patient, the amount of the material that has worn away, and various other things. I think the paper will do good in calling our attention so earnestly to certain points. Sometimes a strong statement will attract our notice, whereas if the same facts were put in a mild way, it would be passed over and not so much attention given to it.

*Dr. Bonwill.*—I hope you will have time enough to hear what I have to say, and particularly for my friend Dr. Lord to discuss anything that may be necessary to settle these questions. With all due respect for the pioneers in dentistry; with all that love we have for the person; with all the magnitude of feeling that we may cultivate, we must throw aside certain things if we are forced to take care of ourselves. I will not be insulted or hurt by anybody, especially by Dr. Lord. I know he does not mean it. I know there are some things that Dr. Lord does not know. We might talk forever upon dental subjects which come up over and over again. You see history repeating itself. I have not attempted to present anything but what I have done in my practice. I do not ask any one to follow me; I do not care whether they follow me or not. I go on my way no matter how many obstructions I meet. I have the consciousness just how nearly I am doing right and wrong. I do not begin even yet to come up to the goal which I would like to reach. I have not said that gold is a failure. There has not been a word in my article or an action in my life that has told you that. I simply would repeat what Dr. Perry has said, that you are indebted to me for the best instruments for the purpose of making more thorough operators. Don't you suppose I love my own children so well that I would not do them any injustice, but for their sake favor the use of gold in every possible case? I have gone into so many offices where the operators did not know how to use my instruments. Each one of us, as Dr. Bogue said the other night, has a peculiar practice. I do not know what Dr. Perry's practice is. But the failures will come. So far as my own practice is concerned, I use gold in every mouth, but I use it less than I did in the large cavities that come to me which are failures of other men. If you gentlemen will simply bring some of your patients, not to show how we can do a thing, but to show how we have done it, it would be better. We can talk as much as we please, every man has his own conclusions. You have no idea of the number or character of his patients. There are people who have been his patients for years. He saves teeth, and he saves them with gold. With all his operations, he does not come up to that standard which we all admit to be perfect. There is not a man but should use a mallet of some kind. No man can stand at the dental chair without using power instruments for the packing of gold. Dr. Lord does not do it.

*I* not love gold? *I* call it a failure? No, it is not so. It is not in the material that the failure comes; it is in the manner in which men fail to recognize certain principles in the preparation of a cavity, in cutting the cavities as far as you can from the centre, making the fullest contour that you can. That is what I do when I tell you to make a plus condition. I tell you how I save so many pulps from destruction. I tell you how I save so many teeth from subsequent caries. I have had some of Dr. Lord's patients, and he has had some of mine. I have had patients of almost every man of prominence in the land. I have come in contact with a larger number of dentists and materials and instruments than any man in the profession. If my friend Dr. Lord would come and stand by my chair and see the immense destruction of the human teeth that comes into my hands, he would say I was doing right. One of the most rabid men in Philadelphia in the use of gold was in my office one day. A patient of mine came in, and in that patient's teeth I had placed some large gold fillings. In the majority of them, however, I put in large amalgam fillings. I said to this dentist, "Will you please tell that patient whether I have done wrong in putting so much amalgam in his mouth?" He looked from every standpoint, and he said, "I do not see how you could have done otherwise."

I still put in immense gold fillings, and Dr. Lord never put in a larger gold filling than I did at a clinic at the University of Pennsylvania, where I used three books of gold in one tooth, and did it in forty-seven and a half minutes. The reason I use so much gutta-percha is to get the tooth-substance as far away as possible. Unless you do that, your work is largely in vain.

There is another point that I wish to speak about that none of you have noticed. You all speak as if you seldom use amalgam, and yet every one of you, I know, uses it, even Dr. Lord. I know just how much amalgam is used by others, and am willing to bring my practice to show you how much good I am doing with it. A dentist has no right to condemn operations performed by another until he knows the peculiar environments around which the operator has had to work. If any man has a right to say what I do about amalgam, it is myself, after I have given you the best instruments known to dentistry for filling with gold. I was told when I was in the West, "Bonwill, don't say anything against gold." Some of them said they would not use my mallet. I asked why, and they said, "If our patients knew we filled teeth in half the time, they would make us charge less." I have been all over the country and learned many good points from some of the men.

Dr. Perry may be my friend. He must know, taking my practice altogether, that I have some right to a following, and I know I have a following; and if I could have Dr. Lord stand by my chair and see my operations, he would say the same thing as other men do, that I am doing a noble and useful work.

I would not recall one word.

It is to be regretted that gentlemen are so very careful what they say about the use of amalgam in their own practice that but few present rise to the discussion, which is not very complimentary, to say the least.

I said all that was necessary in my article, and I thank those gentlemen who have spoken.

A vote of thanks was offered to Dr. Bonwill.

Adjournment.

John I. Hart, D.D.S.,
*Editor New York Odontological Society.*

